# Serum Interleukin 6, Controlling Nutritional Status (CONUT) Score and Phase Angle in Patients with Crohn’s Disease

**DOI:** 10.3390/nu15081953

**Published:** 2023-04-18

**Authors:** Iolanda Cioffi, Filippo Scialò, Olivia Di Vincenzo, Monica Gelzo, Maurizio Marra, Anna Testa, Fabiana Castiglione, Maria Vitale, Fabrizio Pasanisi, Lidia Santarpia

**Affiliations:** 1 Department of Food, Environmental and Nutritional Sciences—DEFENS, Division of Human Nutrition, Università degli Studi di Milano, Celoria 2, 20133 Milan, Italy; 2 Department of Clinical Medicine and Surgery, Federico II University Hospital, Pansini 5, 80131 Naples, Italy; 3 CEINGE—Biotecnologie Avanzate F. Salvatore, s.c.ar.l, 80145 Napoli, Italy; 4 Department of Translational Medical Sciences, University of Campania L. Vanvitelli, 80138 Naples, Italy; 5 Department of Public Health, Federico II University Hospital, Pansini 5, 80131 Naples, Italy; 6 Department of Molecular Medicine and Medical Biotechnology, School of Medicine, University of Naples Federico II, 80131 Naples, Italy

**Keywords:** inflammatory bowel disease, malnutrition, CONUT score, phase angle, c reactive protein

## Abstract

Crohn’s disease (CD) is a chronic inflammatory disorder that may occur in any segment of the gastrointestinal tract. Asymptomatic or untreated inflammation along with malnutrition can often coexist in patients with CD, impairing clinical outcomes, therefore the aim of this study was to assess the relationship between inflammation, malnutrition risk and nutritional status in CD patients. Consecutive adult CD outpatients aged 18-65 years were recruited. Disease activity was clinically defined by the Crohn’s Disease Activity Index (CDAI), whilst anthropometry and phase angle (PhA) were measured. The Controlling Nutritional Status (CONUT) score was retrospectively calculated for screening malnutrition risk and blood samples were taken. A total of 140 CD patients with a mean age of 38.8 ± 13.9 years and an average weight of 64.9 ± 12.0 kg were included. Serum interleukin (IL)-6 concentration was increased in active-CD patients, unrelated to medical treatment, which was associated with CDAI and PhA. Based on the CONUT score, the prevalence of patients with moderate/severe malnutrition risk (score ≥ 5) was 10%, showing lower age, body mass index and fat mass, but higher IL-6 and IL-1β levels than subjects classified as not at risk (score 0–1). Finally, increased IL-6 levels and reduced PhA values were identified as independent predictors of moderate/severe malnutrition risk (*p* < 0.05). In conclusion, IL-6 increased in active-CD patients, which was inversely correlated with PhA. Although the CONUT score might be helpful for identifying CD patients at moderate/severe risk of malnutrition, larger studies are needed to verify these results in different settings.

## 1. Introduction

Crohn’s disease (CD) is a chronic inflammatory disorder characterized by periods of exacerbation and remission that may occur in any segment of the gastrointestinal tract [1,2,3]. The precise CD etiology remains unknown, deriving from a combined effect of genetic susceptibility, environmental factors, and immunity dysregulation [4,5,6].

As it is known, cytokines, mainly produced by monocytes and macrophages in the intestinal wall [7], play a key role in the pathogenesis of the disease, participating in the initiation and perpetuation of inflammatory processes in the gut, which in turns lead to an overbalance of proinflammatory versus anti-inflammatory cytokines, and progressive bowel damage [7]. It is nowadays recognized that early intervention and intensive monitoring of chronic and untreated inflammation, even if asymptomatic, may prevent disease complications [3,8,9]. Among cytokines, interleukin 6 (IL-6) is one of the most prominent due to its pleiotropic effects on inflammation and immunity, which can both boost and reduce inflammation, depending on the target cells [10]. Interleukin-1 beta (IL-1β) is the main factor inducing the production of IL-6. Several studies reported that serum levels of IL-6 increased in patients with disease relapse and decreased during the remission phase, representing a potential biomarker for evaluating the intensity of inflammatory changes in the gastrointestinal tract [6,11,12,13]. 

Besides inflammation, most patients over time tend to develop malnutrition, which can lead to significant impairment of gastrointestinal symptoms, absorption, and intakes of nutrients [14,15,16,17], impacting disease outcomes and quality of life [18]. Thus, screening for and assessing nutritional status is utterly relevant in these patients. Several tools have been adopted so far to establish an effective malnutrition screening tool, such as the Controlling Nutritional Status (CONUT) score, which was first reported for the early detection of hospital malnutrition [19]. The CONUT score is easily calculated from serum albumin level, total blood cholesterol, and total lymphocyte count, but most interestingly, it is recognized to be a promising tool for representing immune-nutritional status [20]. Research in this area showed that the CONUT score has been widely used as a prognostic tool in cancer patients, proving to be an independent risk factor for postoperative complications in patients with hepatocellular carcinoma [20,21]; however, nowadays, only a few studies have been performed in CD patients [22,23]. Recently, Dong et al., 2020 [22] showed that high CONUT scores were linked to low body mass index (BMI) and poor postoperative outcomes in patients with CD undergoing bowel resections, resulting as an independent risk factor for postoperative complications in CD [22]. Therefore, early detection of nutritional risk and adequate monitoring of nutritional status might be crucial for identifying disease exacerbation and administering timely medical treatments. Actually, we recently found that phase angle (PhA), a proxy measure of cellular health and membrane integrity measured by bioelectrical impedance analysis (BIA), represented a valid indicator of nutritional status in CD patients, which is strictly associated with disease activity [24]. 

In light of our previous findings and considering that systemic inflammation and malnutrition often coexist in this population, we looked into the potential associations between levels of pro-inflammatory cytokines, malnutrition risk, screened by the CONUT score and PhA in our cohort of patients with CD [24]. Specifically, we aimed (1) to determine serum concentrations of IL-1β, Il-6 and TNF-α, considering differences according to disease activity and C reactive protein (CRP) levels, (2) to evaluate the prevalence of malnutrition risk using the CONUT score in patients with CD and its relationship with different indicators of nutritional status and inflammatory biomarkers; and eventually, (3) to identify potential predictors of malnutrition risk in patients with CD.

## 2. Materials and Methods

### 2.1. Design and Study Population

This is a retrospective analysis that includes a cohort of consecutive outpatients with CD recruited at the Department of Clinical Medicine and Surgery, Federico II University Hospital, Naples (Italy), between July 2016 and March 2018. All participants signed informed consent prior to enrolment. 

As previously described [24], patients aged between 18 and 65 years and diagnosed with CD were selected. Exclusion criteria were: presence of fistulae, ileostomy, or colostomy; current enteral or parenteral nutrition; residual small bowel <2 m; history of acute or chronic liver or kidney disease; use of corticosteroids in the last 3 months; unstable body weight in the last month; pregnancy or lactation; and unable or unwilling to give informed consent.

Disease activity was clinically defined by the Crohn’s Disease Activity Index (CDAI), classifying patients in the active and quiescent phases (≥150 and <150, respectively). Finally, demographic data, smoking habits, disease duration, medical treatment, previous surgery, location and disease behavior (Montreal classification) were collected.

The protocol of this study was approved by the Federico II Ethical Committee (No. 102/16) and registered on clinicaltrials.gov as NCT03054935. 

### 2.2. Inflammatory Markers 

Serum TNF-α, IL-1β and IL-6 analysis was performed using patients’ blood samples taken early in the morning after an overnight fast. The obtained samples were supplemented with anticoagulant EDTA and stored at −80 °C until the examination. Cytokines were examined by automated microfluidic immunoassay cartridges on ProteinSimple Ella (Bio-Techne^®^ Minneapolis, MN, USA), in accordance with the manufacturer’s instructions. 

C-reactive protein (CRP) (mg/L) was analyzed at the centralized laboratory of Federico II University Hospital following standardized techniques, as previously stated [24]. Then, the following CRP cutoff levels (CRP > 5 mg/L and CRP ≤ 5 mg/L) were used to assess differences in cytokines levels.

### 2.3. Nutritional Screening Risk Tool: CONUT Score

The CONUT score was calculated using serum albumin levels, total lymphocyte count and total cholesterol concentration. A specific score is assigned to each parameter, according to the undernutrition degree [19], and the total CONUT score is obtained by summing each score and classifying patients as having normal (0–1), light (2–4), moderate (5–8) and severe (9–12) risk of malnutrition [19].

### 2.4. Nutritional Assessment 

As reported elsewhere [24], body weight and height were measured to the nearest 0.1 kg and 0.5 cm, respectively, using a platform beam scale with a built-in stadiometer (Seca 709; Seca, Hamburg, Germany). Both measures were taken while the subjects wore light clothes and no shoes. Body mass index (BMI) was calculated as weight (kg) divided by squared height (m^2^). 

Bioelectrical impedance analysis (BIA) [25] was performed using a Human In Plus II device (DS Medica, Milan, Italy) on the nondominant side of the body, with patients lying in supine position for at least 20 min before, starting measurement at a constant room temperature (23–25 °C). BIA-derived phase angle (PhA) was measured at 50 kHz and expressed in degrees. Whilst fat free mass (FFM) and fat mass (FM) were estimated using the predictive equations developed by Kushner [26].

### 2.5. Statistical Analysis 

Descriptive results are presented as mean and standard deviation (SD) or median and ranges or N (%), as appropriate. The Kolmogorov–Smirnov test and the Shapiro–Wilk test were used to check whether variables were normally distributed. General Linear Model was used to test differences in cytokines concentrations according to disease activity (active versus quiescent) and CRP levels (CRP> 5 mg/L vs. CRP ≤ 5 mg/L) using sex and treatments as covariates. Partial correlation analysis was performed using Spearman’s rank coefficient, controlled for sex, to assess the relationship between pro-inflammatory cytokines, nutritional indicators and CONUT score. ANOVA or the Mann–Whitney test with post-hoc comparisons were used as appropriate to assess differences among CONUT score groups. Finally, a logistic regression analysis was used to explore independent risk factors of nutritional status in patients with CD, considering subjects with CONUT score < 5 as the reference group. A *p* value < 0.05 was considered statistically significant. Statistical analyses were performed using the SPSS Statistics software (version 28.0.0, SPSS Inc., Chicago, IL, USA).

## 3. Results

The characteristics of 140 CD patients meeting the inclusion criteria are summarized in Table 1. Mean age of the study group was 38.8 ± 13.9 years and 59% (n = 82) were men. Average body weight and BMI were 64.9 ± 12.0 kg and 23.2 ± 3.72 kg/m^2^, respectively, with 6.4% of patients underweight and 29 (~20%) classified as with overweight/obesity. 

Seventy-eight patients were clinically quiescent (CDAI < 150), while 62 showed mild to moderate disease activity (150 > CDAI < 450). Phenotype of disease was classified according to the Montreal classification, revealing that CD was mostly diagnosed at an age between 17 and 40 years, located in the ileum-colon with a structuring phenotype. Previous surgery due to disease complications was found in half of patients. Regarding the use of medications, 40% of patients were on biologic agents, while 31% were not treated at the time of the visit since they were on the screening phase before starting with biologic therapy.

### 3.1. Cytokines Assessment According to CDAI and CRP Levels

**Figure 1:**
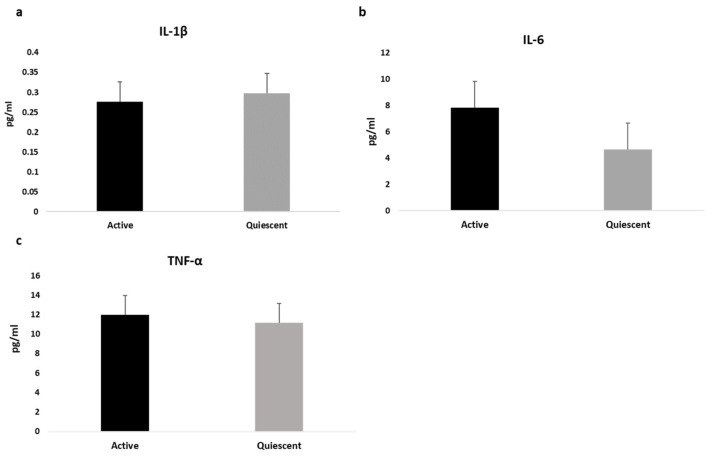
Differences in cytokines values between active and quiescent CD patients, adjusted by sex and treatment. Data are expressed as estimated means and standard error (SE) for Interleukin-1β (**a**), Interlukin-6 (**b**) and Tumor Necrosis Factor-Alfa (**c**).

The following pro-inflammatory cytokines IL-1β, IL-6 and TNF-α were assessed and values for the entire sample as well as divided by sex were reported in Table 1, showing that both IL-1β and Il-6 differed between sexes. Based on CDAI, data showed higher values of cytokines in clinically active subjects compared to quiescent patients (IL-1β = 0.214 pg/mL vs. 0.124 pg/mL, *p* = 0.005; IL-6 = 4.14 pg/mL vs. 2.74 pg/mL, *p* = 0.005; TNF-α = 11.3 pg/mL vs. 9.96 pg/mL, *p* = 0.05; respectively). After sex and treatment adjustment, only serum IL-6 concentration still differed between groups, as shown in Figure 1.

**Figure 2:**
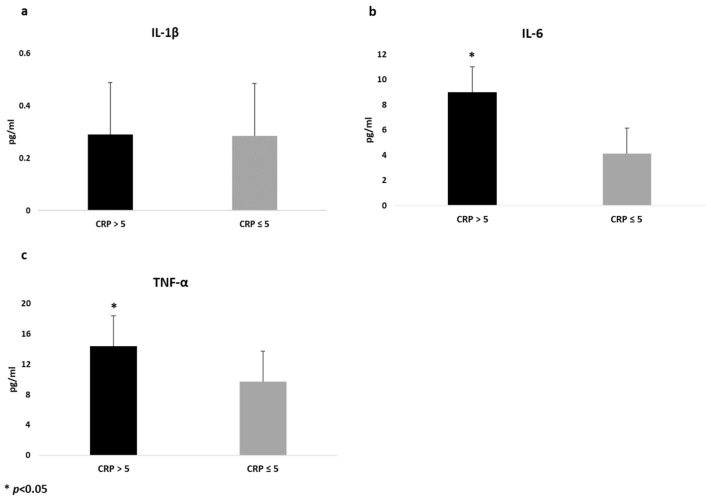
Differences in cytokines values between CD patients with different levels of C reactive protein (CRP), adjusted by sex and treatment. Data are expressed as estimated means and standard error (SE) for Interleukin-1β (**a**), Interlukin-6 (**b**) and Tumor Necrosis Factor-Alfa (**c**).

Similarly, when cytokines were analyzed according to CRP levels (≤5 mg/dL versus >5 mg/dL), we found that patients with CRP > 5mg/dL (n = 55) showed higher levels of IL-6 (9.01 (1.15) pg/mL versus 4.16 (0.92) pg/mL; *p* = 0.001) and TNF-α (14.3 (0.87) pg/mL versus 9.72 (0.69) pg/m, *p* = 0.001) compared to those with lower CRP (≤5 mg/dL), even after sex and treatment adjustment, whilst no difference was found on IL-1 β, as shown in Figure 2.

#### Correlation Coefficients between Cytokines, CRP and Nutritional Indicators

Data from Spearman correlation coefficients, controlled by sex, evaluating the association of serum cytokines with individual characteristics, BIA variables and CRP in CD patients, are presented in Table S1. Serum IL-6 was inversely correlated with FFM (r = −0.190; *p* = 0.02) and PhA (r = −0.184; *p* = 0.03), while it was positively associated with CDAI (r = 0.296; *p* = 0.001), CRP (r = 0.285; *p* = 0.001) and CONUT score (r = 0.270; *p* = 0.001). On the other hand, neither IL-1β nor TNF-α were associated with nutritional parameters, except for CPR which displayed a positive correlation with TNF-α (r = 0.450; *p* = 0.001).

### 3.2. CONUT Score

**Figure 3:**
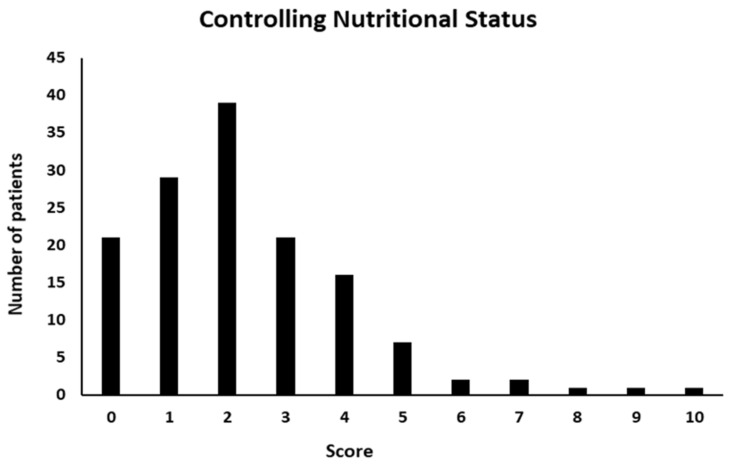
Controlling Nutritional Status (CONUT) score distribution.

According to CONUT, the median score was 2 (range, 0–10). Among 140 CD patients, 90 were found to be at risk of malnutrition, of which 50 (66%) were men. In details, 76 (55%) were at light risk, 12 (8%) at moderate and only 2 (1%) at severe (score: 9–12) risk of malnutrition, whereas 50 (36%) patients were classified as normal or not at risk (Figure 3).

Considering that CONUT score ≥ 5 was commonly defined as the cut-off for a significant risk of malnutrition, patients were split into three groups as follows: CONUT 0–1 (normal or no risk), CONUT 2–4 (light risk) and CONUT ≥ 5 (moderate/severe risk), to detect differences in both nutritional indicators and cytokines concentrations based on nutritional classification.

#### 3.2.1. Comparison of CDAI and Nutritional Indicators in CD Patients According to CONUT Score

CDAI, age and nutritional variables according to the three CONUT score groups were compared, as presented in Table 2. Findings revealed that patients at risk of malnutrition (CONUT ≥ 2) were younger and had a lower BMI than those identified as not at risk (CONUT 0–1). Only patients with higher CONUT scores (≥5) showed a significant decrease in both absolute and percentage FM values compared to subjects from the other two groups. Similarly, a progressive decline in PhA values, albeit not statistically significant (*p* = 0.07), was found in patients with a high CONUT score. No difference was observed for CDAI, weight and FFM, instead.

#### 3.2.2. Comparison of Inflammatory Markers in CD Patients According to CONUT Score

The following biomarkers CRP, IL-1β, IL-6 and TNF-α were assessed and compared among the three CONUT score groups (Table 3). Results showed that CRP differed only between patients not at risk and those at moderate/severe risk (CONUT 0–1: 2.0 (4.9) mg/L, versus CONUT ≥ 5: 8.5 (27.1) mg/L; *p* = 0.008); while higher levels of both IL-1 β and IL-6 were found in patients at high risk of malnutrition (CONUT ≥ 5) compared to those at low risk. No difference among groups was found in TNF-α values.

### 3.3. Predictors of Malnutrition Risk Based on CONUT Score

Logistic regression analysis was used to assess potential independent predictors of malnutrition risk in CD patients defined by CONUT score. Data identified reduced PhA values (odds ratio [OR] = 0.296 95%CI 0.093 to 0.946, *p* = 0.04) and increased levels of serum IL-6 (OR = 1.165 95%CI 1.028 to 1.321; *p* = 0.02) as independent predictors of moderate/severe risk of malnutrition, as shown in Table 4.

## 4. Discussion

This retrospective analysis aimed to assess the relationship between inflammation, malnutrition risk and nutritional status in patients with CD, using cytokines concentrations, CONUT score and PhA, respectively. Overall, serum IL-6 concentration was increased in active CD patients as well as in patients with CRP levels > 5 mg/dL, which was positively associated to CDAI and CRP, but negatively with FFM and PhA. According to the CONUT score, the prevalence of moderate/severe risk of malnutrition (score ≥ 5) in CD patients was 10%. This group of patients showed lower age, BMI and FM values, but higher CRP, IL-6 and IL-1β levels compared to those at no risk. Interestingly, reduced PhA values and increased IL-6 levels were identified as independent predictors of moderate/severe risk of malnutrition, suggesting that the CONUT score might be a reliable tool for identifying patients at high risk of malnutrition.

Generally, given the chronic nature of inflammation in CD, often characterized by a disconnection between clinical symptoms and underlying inflammation, it is of vital importance to monitor disease at regular intervals, also involving different health care maintenance (nutritional deficiencies, quality of life, etc.) using objective and measurable indicators [3,27]. Regarding the inflammatory status, CRP is one of the most widely used serum markers of inflammation in inflammatory bowel disease (IBD), but it is not disease specific, since elevated levels occur in several inflammatory disorders not related to the gastrointestinal tract [28].

Recently, a greater attention has been paid to the pleiotropic effect of cytokine IL-6, which plays an important role in inflammatory diseases [5]. We found higher concentrations of serum IL-6 in active versus quiescent CD patients but also in the group of individuals with CRP levels > 5mg/dL, unrelated to medical treatment. Furthermore, IL-6 showed a strong correlation with CRP. In fact, IL-6 promotes T-cell population expansion and activation, B-cell differentiation and regulates the acute phase response [29,30,31], being the most important stimulus for the synthesis of CRP [32]. Physiological concentrations of IL-6 in human serum are relatively low, but during inflammatory conditions, they increase rapidly and earlier than CRP. Moreover, IL-6 levels have been described to predict disease activity better than CRP [29]. Previous works reported an overall increase in IL-6 levels in patients with IBD, particularly in those with active disease [33,34,35]. More recently, Nikolaus et al. [5] highlighted increased serum levels of IL-6 in active versus non-active CD patients, also compared with healthy controls, reporting an association between IL-6 and C-reactive protein concentrations, in line with our current findings. In addition, the combination of high concentrations of both IL-6 and CRP showed a higher diagnostic accuracy than each parameter taken alone [29]. Indeed, CRP level was more strongly associated to elevated IL-6 levels than score-based activity (CDAI), likely due to the key role of cytokine in CRP secretion [5] and considering that CDAI is just a clinical index of disease activity.

CRP was also found to be correlated to TNF-α concentration in the present study, with the latter increasing consistently in the group of patients with CRP levels ≥ 5 mg/dL, even after treatment adjustment. Accordingly, a previous study showed no significant difference in serum TNF-α concentrations in CD patients related to disease activity [36].

As a general biomarker of disease activity in IBD, IL-6 adds little to recognized biomarkers like CRP, in line with previous findings [5,33,34], considering that IL-6 is not routinely measured in the clinical setting, its standardization is low and cost is definitely higher than CRP; but it might be potentially linked to nutritional status.

Indeed, malnutrition is definitely another crucial issue in CD patients due to its capacity to affect CD outcomes. Hence, an early identification and continuous monitoring can provide valuable information in clinical practice [37]. Therefore, identifying patients at nutritional risk by validated screening tools is the first step to fulfil prior to perform nutritional assessment [38,39]. Since inflammation has a remarkable role in the pathogenesis of malnutrition, it is important to evaluate disease-related malnutrition with or without inflammation [37]. We explored the association between inflammatory markers, CONUT score and nutritional indicators among CD patients, observing that IL-6 displayed a positive association with CONUT score, while it was inversely correlated to PhA and FFM.

Different tools for nutritional screening have been used in the clinical practice so far. Among them, the CONUT score has become popular because of the easiness of retrieving laboratory data appropriate for retrospective analysis [40]. Both inflammatory and nutritional status parameters used for its calculation rely on serum and/or blood counts in peripheral blood, which are routinely measured in daily clinical practice [41,42,43]. In the last years, the CONUT score has shown to be an independent predictor associated with prognosis in patients with different conditions, such as cancer, cardiovascular and cerebrovascular diseases [21,39,43]; however, its use in CD patients or IBD cohorts has been still poorly explored.

Our findings highlighted that patients with a high CONUT score were younger and had low BMI, FM values but increased levels of CRP and IL-6. Previously, Dong et al. [22] used CONUT score to explore its association with postoperative complications in CD patients, revealing that high CONUT scores were found in patients with lower BMI and its predictive accuracy was better than that of albumin alone or other nutritional screening tools. In the present study, the CONUT score was applied to a population of medical, non-complicated CD patients, in different phases of the disease. Recently, the CONUT score has been compared to other nutritional indexes in acute care patients, substantially observing that it was much sensitive in patients with more inflammation [44]. Accordingly, both CRP, IL-1β and IL-6 were considerably increased in patients with a high CONUT score compared to those with a low CONUT score, highlighting its role in predicting immune status. Since inflammation is a remarkable driving force for disease-related anorexia, decreased food intake and muscle catabolism, regardless of the underlying disease, the use of the CONUT score, which has been shown to evaluate contemporarily immune status, protein reserve and lipid metabolism, and to predict mortality in many inflammatory diseases [20,41,42], might be useful in this population.

Finally, in this sample, IL-6 levels increased with decreasing PhA values, and both parameters were meaningful predictors of moderate/severe malnutrition risk. Among nutritional variables, PhA has recently gained great popularity in nutritional assessment and monitoring in different disease conditions [24,45,46]. Indeed, PhA was recently found to be a valid indicator of disease activity and nutritional status in CD patients by our group and others [47]. The inverse association observed between IL-6 and PhA might be at least partly explained by the fact that prolonged inflammatory processes can adversely impact tissue hydration and vascular/cellular permeability, affecting tissue electrical properties and resulting in significant decreases of PhA values compared with healthy subjects [48]. Indeed, reactive oxygen species disrupt cell membranes and fluid balance between intracellular and extracellular spaces, altering the capacitive effect of membranes and consequently, PhA [49]. Accordingly, the negative relationship between IL-6 and PhA was also observed in different type of patients as reported by a recent review about PhA and inflammation [50]. Finally, in this study, we observed a progressive decline, albeit not statistically significant (*p* = 0.070), of PhA and the concomitant increase of the CONUT score (≥5), suggesting that PhA can potentially detect malnutrition risk.

Notably, the negative effects of both malnutrition and inflammation were observed on body composition, especially on FFM. In this study, however, FFM has been estimated by BIA and this might be considered a limitation for several reasons, let alone the lack of specific equations for estimating FFM in CD patients. Generally, errors in FFM estimation from BIA derive from errors in total body water (TBW) estimation, as a consequence, lower TBW values (e.g., dehydration) yield underestimates of FFM; while overhydration results in overestimation, as it potentially occurred in this sample, when looking at differences in FFM among CONUT score groups. Therefore, these current results need to be cautiously interpreted.

### 4.1. Strengths and Future Perspectives

To our knowledge, this is the first study exploring the relationship between IL-6, CONUT and PhA in patients with CD. The strengths of this current analysis are to show the potential application of the CONUT score to assess malnutrition risk in a large group of patients with CD, and also its good correlation with both nutritional and inflammatory status of patients, highlighting its prognostic value when associated with PhA and IL-6 levels.

Indeed, given the coexistence and reciprocal influence of inflammation and malnutrition risk in these patients, the use of the CONUT score, in combination with other markers such as PhA and/or IL-6, could be a relevant tool for a more rapid and reliable assessment of patient’s status and disease outcomes in the clinical setting. Eventually, the assessment of additional serum biomarkers, even more specific than IL-6, linked to the inflammatory response, might provide new insights into the management of CD patients.

### 4.2. Limitations

However, several limitations need to be acknowledged. First, this is a retrospective analysis performed in a specific selected sample of patients with CD, and probably not fully representative of the disease. Second, even though nutritional screening tools, such as the CONUT score, based on laboratory parameters are objective, reproducible, and timesaving, allowing for an automatic assessment, it is important to verify that the serum parameters involved are not significantly affected by the severity of disease. Last but not least, larger prospective studies are needed to verify the feasibility and reliability of these data in different settings.

## 5. Conclusions

In conclusion, serum IL-6 increased in active CD patients, unrelated to treatment, which was positively correlated with CDAI and CRP, but negatively with PhA. Apparently, the CONUT score could be helpful for identifying CD patients with moderate/severe malnutrition risk (CONUT ≥ 5), showing that reduced PhA values and increased IL-6 levels emerged as independent predictors of malnutrition risk. However, larger prospective studies are needed to explore the link between inflammation and malnutrition in larger groups of CD patients, focusing on the clinical use of the CONUT score.

## Figures and Tables

**Table 1:** Demographic and clinical characteristics of CD patients.

	Total	Men	Women
N, (%)	140 (100)	82 (58.6)	58 (41.4)
Age, y (mean ± SD)	38.8 ± 14.0	38.1 ± 13.8	39.9 ± 14.4
Body weight, kg (mean ± SD)	64.9 ± 12.0	69.8 ± 10.2	58.0 ± 11.1 **
BMI, n (%)			
<18.5 kg/m^2^	9 (6.4)	1 (1.2)	8 (13.8)
18.5–24.9 kg/m^2^	102 (72.9)	66 (80.5)	36 (62.1)
25-29.9 kg/m^2^	21 (15)	12 (14.6)	9 (15.5)
>30 kg/m^2^	8 (5.7)	3 (3.7)	5 (8.6)
BIA variables, mean ± SD			
FFM, kg	49.2 ± 10.2	56.0 ± 6.47	39.6 ± 5.86 **
FM kg	15.7 ± 8.10	13.7 ± 7.43	18.5 ± 8.24 **
FM, %	23.9 ± 10.4	19.0 ± 8.36	30.9 ± 8.93 **
PhA, degrees	6.36± 0.94	6.80 ± 0.89	5.72 ± 0.59 **
CRP, mg/L, median (IQR)	3.15 (8.0)	3.1 (9.4)	3.1 (7.1)
Cytokines, pg/L, median (IQR)			
IL-1β	0.16 (0.25)	0.13 (0.27)	0.18 (0.21) *
IL-6	3.47 (5.8)	3.84 (6.1)	3.15 (3.0) *
TNF-α	10.4 (4.7)	10.3 (4.2)	10.4 (5.3)
Previous surgery, n (%)	74 (52.9)	41 (50.0)	33 (56.9)
Mean duration, y [median; range]	8.80 [0.5–36]	9.01 [1–30]	8.52 [0.5–36]
Clinical activity, n (%)			
CDAI < 150	78 (55.7)	50 (61)	28 (48.3)
>150 CDAI <450	62 (44.3)	32 (39)	30 (51.7)
Montreal age at diagnosis, n (%)			
A1: <16 y	26 (18.6)	17 (20.7)	9 (15.5)
A2: 17–40 y	93 (66.4)	53 (64.6)	40 (69.0)
A3: >40 y	21 (15.0)	12 (14.6)	9 (15.5)
Montreal disease location, n (%)			
L1: Ileum	46 (32.9)	28 (34.1)	18 (31.0)
L2: Colon	11 (7.9)	10 (12.2)	1 (1.7)
L3: Ileum and colon	80 (57.1)	42 (51.2)	38 (65.5)
L4: Upper GI tract	3 (2.1)	2 (2.4)	1 (1.7)
Montreal disease behaviour, n (%)			
B1: Inflammatory	37 (26.4)	27 (32.9)	10 (17.2)
B2: Stricturing	76 (54.3)	43 (52.4)	33 (56.9)
B3: Penetrating	27 (19.3)	12 (14.6)	15 (25.9)
Medications, n (%)			
None	43 (30.7)	23 (28.0)	20 (34.5)
5-ASA	24 (17.1)	14 (17.0)	10 (17.2)
IMMs	17 (12.1)	9 (17.1)	8 (13.8)
Biologics	56 (40.0)	36 (43.9)	20 (34.5)

A: Age at diagnosis; ASA: Amino Salicylic Acid; B: Disease Behavior; BMI: Body Mass Index; CDAI: Chron’s Disease Activity Index; CRP: C-Reactive Protein; FFM: Fat Free Mass; FM: Fat Mass; IL-1β: Interleukin-1beta; IL-6: Interleukin-6; IMMs: Immunosuppressives; IQR: Interquartile Range; L: disease Location; SD: Standard Deviation; TNF-α: Tumor Necrosis Factor-alfa; y: year. Sex differences: * *p* < 0.05; ** *p* < 0.001.

**Table 2:** Comparisons of CDAI, age and nutritional parameters in CD patients according to CONUT score groups.

	CONUT 0–1	CONUT 2–4	CONUT ≥ 5	*p*
	(n = 50)	(n = 76)	(n = 14)	
CDAI	125 ± 74	140 ± 79	172 ± 99	0.132
Age, y	45.2 ± 12.9 ^	35.4 ± 13.5	34.1 ± 12.4	0.000
Weight, kg	66.4 ± 13.7	64.8 ± 11.4	60.1 ± 6.37	0.237
BMI, kg/m^2^	24.5 ± 4.31 ^	22.8 ± 3.21	20.5 ± 1.94	0.001
FFM, kg	47.0 ± 9.98	50.0 ± 10.6	52.5 ± 8.16	0.122
FM, kg	19.3 ± 8.63	14.8 ± 6.96	7.72 ± 3.99 ‡	0.000
FM, %	28.6 ± 9.61	22.8 ± 9.60	13.1 ± 7.60 ‡	0.000
PhA, degrees	6.22 ± 0.80	6.51 ± 0.98	5.97 ± 1.09	0.070

Data are expressed as mean ± standard deviation. BMI: Body Mass Index; CD: Chron’s Disease; CDAI: Chron’s Disease Activity Index; CONUT: Controlling Nutritional Status; FFM: Fat Free Mass; FM: Fat Mass; PhA: Phase Angle; y: years. ANOVA test and Bonferroni post-hoc comparisons between groups: ^ CONUT 0–1 vs. CONUT 2–4 and CONUT ≥ 5; ‡ CONUT ≥ 5 versus CONUT 0–1 and CONUT 2–4.

**Table 3:** Comparisons of CRP and cytokines in CD patients according to CONUT score.

	CONUT 0–1	CONUT 2–4	CONUT ≥ 5	*p*
	(n = 50)	(n = 76)	(n = 14)	
CRP, mg/L	2.0 (4.9)	3.1 (10.2)	8.5 (27.1) §	0.008
IL-1β, pg/mL	0.13 (0.22)	0.15 (0.25)	0.33 (0.47) ‡	0.008
IL-6, pg/mL	2.22 (3.4)	3.55 (4.4)	9.83 (12.9) ‡	0.000
TNF-α, pg/mL	9.88 (5.1)	10.8 (4.5)	11.8 (5.5)	0.219

Data are expressed as median and interquartile range. CD: Chron’s Disease; CONUT: Controlling Nutritional Status; CRP: C-Reactive Protein; IL-1β: Interleukin-1beta; IL-6: Interleukin-6; TNF- α: Tumor Necrosis Factor-Alfa. Kruskal Wallis test and multiple post-hoc comparisons: § CONUT ≥ 5 vs. CONUT 0–1; ‡ CONUT ≥ 5 versus CONUT 0–1 and CONUT 2–4.

**Table 4:** Logistic regression analysis of predictors of malnutrition risk based on CONUT score.

	OR	95° CIs	*p*
Age, y	0.939	0.869–1.014	0.108
BMI, kg/m^2^	0.712	0.366–1.386	0.318
FFM, kg	1.039	0.852–1.267	0.707
FM, kg	0.783	0.588–1.042	0.094
PhA, degrees	0.296	0.093–0.946	0.040
IL-1β, pg/mL	0.288	0.010–8.641	0.473
IL-6, pg/mL	1.165	1.028–1.321	0.017
TNF-α, pg/mL	0.841	0.659–1.074	0.165
CRP, mg/dL	1.015	0.973–1.059	0.482

BMI: Body Mass Index; CIs: Confidence Intervals; CRP: C-Reactive Protein; FFM: Fat Free Mass; FM: Fat Mass; IL-1β: Interleukin-1beta; IL-6: Interleukin-6; OR: Odds Ratio; PhA: Phase Angle; TNF-α: Tumor Necrosis Factor-Alfa; y: year.

## Data Availability

The data presented in this study are available on request from the corresponding author. The data are not publicly available due to privacy.

## Supplementary Materials

The following supporting information can be downloaded at: https://www.mdpi.com/article/10.3390/nu15081953/s1, Table S1: Spearman’s correlation coefficients between cytokines, CDAI and nutritional indicators in CD patients, after sex adjustment.
