# Supplementary material for: Serum Interleukin 6, Controlling Nutritional Status (CONUT) Score and Phase Angle in Patients with Crohn’s Disease
Source: Nutrients. 2023 Apr 18;15(8):1953. doi: 10.3390/nu15081953 (PMC10146872; doi:10.3390/nu15081953)
Supplement: Supplementary file 1 [file nutrients-15-01953-s001.zip › nutrients-2317570-supplementary.pdf]

Table S1. Spearman's correlation coefficients between cytokines, CDAI and nutritional indicators in CD patients, after sex adjustment.

	IL-1β	IL-6	TNF-α
CDAI	0.041	0.296**	0.097
CRP, mg/dl	0.035	0.285**	0.450**
Age, y	0.010	-0.084	-0.085
BMI, kg/m^2	-0.068	-0.114	-0.032
FFM, kg	-0.107	-0.190*	-0.034
FM, kg	-0.046	-0.036	-0.061
FM, %	-0.024	0.016	-0.003
PhA, degrees	-0.110	-0.184*	-0.026
CONUT score	0.010	0.270**	0.077

**p<0.01; *p<0.05

CD: Chron's disease; CDAI: Chron's Disease Activity Index; CRP:C-reactive protein; y=years; BMI: Body Mass Index; FFM: fat free mass; FM: fat mass; PhA: phase angle; CONUT: Controlling Nutritional Status; ; IL-1β: interleukin-1beta; IL-6: interleukin-6; TNF-α: tumor necrosis factor-alfa.
